# Validation of RESP and PRESERVE score for ARDS patients with pumpless extracorporeal lung assist (pECLA)

**DOI:** 10.1186/s12871-020-01010-0

**Published:** 2020-05-02

**Authors:** Jan Petran, Thorsten Muelly, Rolf Dembinski, Niklas Steuer, Jutta Arens, Gernot Marx, Ruedger Kopp

**Affiliations:** 1 Department of Intensive Care Medicine, Medical Faculty, RWTH Aachen University, Pauwelsstr 30, 52074 Aachen, Germany; 2 Department of Anaesthesiology and Intensive Care Medicine, St. Antonius Hospital, Dechant-Deckers-Straße 8, 52249 Eschweiler, Germany; 3 Clinic for Intensive Care and Emergency Medicine, Bremen-Mitte Hospital, Sankt-Jürgen-Straße 1, 28205 Bremen, Germany; 4 Department of Cardiovascular Engineering, Institute of Applied Medical Engineering, Medical Faculty, RWTH Aachen University, Pauwelsstr 20, 52074 Aachen, Germany; 5 Department of Biomechanical Engineering, Faculty of Engineering Technology, University of Twente, Horst Complex, 7500 AE Enschede, Netherlands

**Keywords:** Acute respiratory distress syndrome, Extracorporeal membrane oxygenation, Pumpless extracorporeal lung assist, Extracorporeal carbon dioxide removal, SOFA score, RESP score, PRESERVE score

## Abstract

**Background:**

RESP score and PRESERVE score have been validated for veno-venous Extracorporeal Membrane Oxygenation in severe ARDS to assume individual mortality risk. ARDS patients with low-flow Extracorporeal Carbon Dioxide Removal, especially pumpless Extracorporeal Lung Assist, have also a high mortality rate, but there are no validated specific or general outcome scores. This retrospective study tested whether these established specific risk scores can be validated for pumpless Extracorporeal Lung Assist in ARDS patients in comparison to a general organ dysfunction score, the SOFA score.

**Methods:**

In a retrospective single center cohort study we calculated and evaluated RESP, PRESERVE, and SOFA score for 73 ARDS patients with pumpless Extracorporeal Lung Assist treated between 2002 and 2016 using the XENIOS iLA Membrane Ventilator. Six patients had a mild, 40 a moderate and 27 a severe ARDS according to the Berlin criteria. Demographic data and hospital mortality as well as ventilator settings, hemodynamic parameters, and blood gas measurement before and during extracorporeal therapy were recorded.

**Results:**

Pumpless Extracorporeal Lung Assist of mechanical ventilated ARDS patients resulted in an optimized lung protective ventilation, significant reduction of P_aCO2_, and compensation of acidosis. Scoring showed a mean score of alive versus deceased patients of 3 ± 1 versus − 1 ± 1 for RESP (*p < 0.01*), 3 ± 0 versus 6 ± 0 for PRESERVE (*p < 0.05*) and 8 ± 1 versus 10 ± 1 for SOFA (*p < 0.05*). Using receiver operating characteristic curves, area under the curve (AUC) was 0.78 (95% confidence interval (CI) 0.67–0.89, *p < 0.01*) for RESP score, 0.80 (95% CI 0.70–0.90, *p < 0.0001*) for PRESERVE score and 0.66 (95% CI 0.53–0.79, *p < 0.05*) for SOFA score.

**Conclusions:**

RESP and PRESERVE scores were superior to SOFA, as non-specific critical care score. Although scores were developed for veno-venous ECMO, we could validate RESP and PRESERVE score for pumpless Extracorporeal Lung Assist. In conclusion, RESP and PRESERVE score are suitable to estimate mortality risk of ARDS patients with an arterio-venous pumpless Extracorporeal Carbon Dioxide Removal.

## Background

Specific mortality risk scores, especially the Respiratory ECMO Survival Prediction (RESP) score [1] and the PRedicting dEath for SEvere ARDS on VV-ECMO (PRESERVE) score [2], were developed and validated for ARDS patients with veno-venous high-flow Extracorporeal Membrane Oxygenation (ECMO). ARDS with severe hypercapnia without life-threatening hypoxemia can be treated with Extracorporeal Carbon Dioxide Removal (ECCO_2_R), especially pumpless Extracorporeal Lung Assist (pECLA). Despite a high mortality rate validated risk scores are lacking for these devices.

During the past decade, ECMO was frequently used for patients suffering severe hypoxemic ARDS, indicated by a Horowitz index PaO_2_/FiO_2_ below 50–80 mmHg despite lung protective ventilation, to maintain gas exchange and facilitate lung protection [3]. In ARDS patients with severe hypercapnia and respiratory acidosis without life-threatening hypoxemia ECCO_2_R was propagated to achieve lung protective ventilation [4]. Arterio-venous pECLA represents a specific subgroup of ECCO_2_R using a simplified extracorporeal lung assist technique for patients with hypercapnia and respiratory acidosis without cardiac failure. It demonstrated efficient extracorporeal carbon dioxide elimination resulting in lung protective ventilation without respiratory acidosis [5] and reducing the risk of ventilator induced lung injury (VILI) [5–7]. pECLA therapy is limited by a low oxygen transfer with only moderate increase of oxygenation.

High mortality rates of ECMO and allocation of limited ECMO resources were leading to the development of mortality prediction scores for veno-venous ECMO in severe ARDS. Especially the RESP score [1] and the PRESERVE score [2] have been used to identify risk factors for death of ECMO patients (additional files 1 and 2). Additionally, non-ARDS-specific scores have been used in critical care. The Sequential Organ Failure Assessment (SOFA) score, published in 1996, evaluates morbidity by scoring the organ failure of lung, coagulation, liver, cardiovascular system, brain, and kidney (additional file 3) [8]. In the prospective observational LUNG SAFE study SOFA score was associated with outcome of ARDS [9]. RESP and/or PRESERVE scores have been compared and evaluated in several studies for ECMO therapy [10–16], but both scores as well as SOFA score have not been validated for ARDS patients treated with a primary extracorporeal CO_2_ removal, like pECLA.

In this retrospective study we tested the hypothesis that RESP and PRESERVE score are suitable to assume the mortality risk of pECLA therapy in case of ARDS and are superior to the SOFA score, which is not specific for Extracorporeal Lung Support and ARDS.

## Methods

We conducted a retrospective single center cohort study of ARDS patients undergoing pECLA therapy between 2002 and 2016 at RWTH Aachen University Hospital to validate RESP, PRESERVE and SOFA score. General ethical approval was received by the RWTH Aachen University regional research ethics committee for retrospective studies and confirmed for this retrospective study (AF 047/16). Inclusion criteria were ARDS according to the Berlin criteria [17] with pECLA therapy and exclusion criteria missing data necessary for calculation of scores.

Standard therapy included a lung protective ventilation strategy with a pressure controlled ventilation mode, usually Biphasic Positive Airway Pressure ventilation: Additionally prone position was initiated in moderate to severe ARDS and inhaled nitric oxide was used as rescue therapy in hypoxemia according to the local standard [18]. In our institution, indication for pECLA and ECMO is confirmed multidisciplinary by physicians of all involved medical faculties. In case of severe hypoxemia due to ARDS indicated by persistent PaO_2_/FiO_2_ < 60 mmHg despite optimized conservative therapy, patients were treated with veno-venous ECMO as rescue therapy. An indication for pECLA was a severe hypercapnia especially in case of concomitant respiratory acidosis (pHa > 7.2 and/or PaCO_2_ > 60 mmHg) as well as achievement of lung protective ventilation, especially when plateau pressure was more than 30 mbar despite optimization of conservative ARDS therapy. The pECLA consisted of a polymethylpentene oxygenator with heparin coating and a membrane surface area of 1.3 m^2^ (iLA Membrane Lung®, Xenios AG, Heilbronn, Germany). Filling volume was 250 ml. The cannulas were inserted in the femoral artery (13 or 15 Fr) and in the femoral vein (15 or 17 Fr). pECLA initiation and therapy was performed according to the manufacturer’s instructions of use and local standards.

The collected data contained origin of ARDS at ICU admission, demographic parameters such as age, sex, height, weight, diseases, hours of ventilation before pECLA initiation, and SOFA score before pECLA. Furthermore, subjects were retrospectively classified in PRESERVE and RESP scores according to the work of Schmidt et al. [1, 2]. We recorded ventilator settings with airway pressures (peak/plateau inspiratory pressure, PEEP, driving pressure) and tidal volume. As all patients were ventilated in a pressure controlled mode peak inspiratory pressure and plateau pressure were equal. Registered hemodynamic parameters were mean arterial pressure (MAP), central venous pressure, heart rate, and norepinephrine dose per minute, and additionally, blood gas measurement with Horowitz index (P_aO2_/F_IO2_), P_aCO2_, pH, and S_aO2_. All parameters were registered straight before pECLA initiation, as well as 2 and 24 h after pECLA initiation. Calculating the scores required specific additional information, such as laboratory values, organ function, comorbidity, medication, and specific interventions before pECLA initiation. Hospital mortality rate was recorded according to the development of RESP Score by Schmidt et al. [1].

For statistical analysis, data are presented as mean and standard deviation (mean ± SD). After confirmation of normal distribution with the Kolmogorov–Smirnov test, significance was tested within groups with repeated-measures ANOVA with post-test and between groups with unpaired t-test (InStat version 3.06, GraphPad, San Diego, CA, USA). A value of *p < 0.05* was considered statistically significant. A multivariable regression analysis including a variable selection assessed the correlation with mortality. With GraphPad Prism 7 (GraphPad, San Diego, CA, USA) receiver operating characteristic (ROC) curves of the scores were calculated and an optimum threshold was defined by calculating the maximum Youden index (J = Sensitivity + Specifity - 1).

## Results

Between 2002 and 2016 79 ARDS patients were treated with pECLA at RWTH Aachen University Hospital. After retrospective screening six patients were excluded due to missing data and 73 subjects were included in the study. Table 1 presents demographic data including severity and origin of ARDS as well as morbidity before pECLA in detail. Thirteen subjects had an immunocompromised status with a significantly higher mortality rate of 85%, defined as hematologic malignancies, solid tumor, solid organ transplantation, human immunodeficiency virus, or liver cirrhosis. All subjects fulfilled the ARDS criteria including a PEEP of at least 5 cm H_2_O according to the Berlin definition [17]. Most patients had a moderate ARDS (Table 1). Fifty-two patients had a severe hypercapnia with a P_aCO2_ ≥ 60 mmHg and 28 a severe acidosis with a pH < 7.2. All subjects were sedated and invasive mechanically ventilated in a pressure controlled mode with a shorter duration before pECLA in the survivor group. During pECLA all patients received invasive mechanical ventilation.

**Table 1:** Patient characteristics before pECLA initiation for total number of patients and subgroup for survival/non-survival to hospital discharge

Characteristics, n (%)	Total	Survivor	Non-Survivor	Mortality
n=	73	37	36	49%
female sex	28 (38)	18 (49)	10 (28)	26%
male sex	45 (62)	19 (51)	26 (72)	58%
age, years	51 +/− 17	44 +/−15*	57 +/− 16*	
Body mass index, kg/m^2^	27.6 +/− 6.1	28.2 +/− 6.8	27.0 +/− 5.2	
SOFA score	9 +/− 3	8 +/− 3^†^	10 +/− 4^†^	
immunocompromised status	13 (18)	2 (5)	11 (31)	85%^‡^
Immunocompetent status	60 (82)	35 (95)	25 (69)	42%^‡^
**Origin of ARDS, n (%)**				
pneumonia	44 (60)	25 (68)	19 (53)	43%
viral	10 (14)	7 (19)	3 (8)	30%
bacterial	29 (40)	14 (38)	15 (42)	52%
aspiration	3 (4)	3 (8)	0 (0)	0%
trauma and burn	10 (14)	5 (14)	5 (14)	50%
status asthmaticus	3 (4)	2 (5)	1 (3)	33%
other	16 (22)	5 (14)	11 (31)	69%
**Severity of ARDS, n (%)**				
mild	6 (8)	4 (11)	2 (6)	33%
moderate	40 (55)	20 (54)	20 (56)	50%
severe	27 (37)	13 (35)	14 (39)	52%
**Ventilator/pECLA therapy**				
Duration of mechanical ventilation before pECLA, days	8 +/− 8	6 +/− 7^†^	10 +/− 10^†^	
Duration of pECLA therapy, days	8 +/− 8	7 +/− 5	9 +/− 9	
rescue therapy (ECMO)	9 (12)	5 (14)	4 (11)	44%
no rescue therapy	64 (78)	32 (86)	32 (89)	50%
**Rescue therapy before pECLA**				
Inhaled nitric oxide	11 (15)	7 (19)	4 (11)	25%
neuromuscular blockade agents	0 (0)	0 (0)	0 (0)	
prone position	10 (14)	4 (11)	6 (17)	60%
**Age, years, n (%)**				
18–49	31 (43)	22 (59)	9 (25)	29%^‡^
50–59	14 (19)	8 (22)	6 (17)	43%^‡^
≥ 60	28 (38)	7 (19)	21 (58)	75%^‡^

Data presented as mean ± SD or number (n) with percent of all patient within the group (%) and hospital mortality of the group, where applicable. * *p < 0.01* alive vs. dead, ^†^*p < 0.05* alive vs. dead, ^‡^*p < 0.01* between groups

Overall hospital mortality rate was 49%, but demonstrated significant age-related differences. Subjects who died in hospital were significantly older and SOFA score was higher before initiation of pECLA. Main Causes of death were septic shock with multi organ failure (44%), non-infectious multi organ failure (17%) and persistent respiratory failure (28%). 11% died due to infaust neurologic prognosis (3 severe head injury after trauma and 1 intracranial bleeding under anticoagulation).

Ventilation, oxygenation, acid-base status, and hemodynamics are presented before initiation of pECLA, after 2 and after 24 h in Table 2. After starting pECLA therapy a significant reduction of inspiratory pressure and driving pressure was observed in all subjects. After 2 and 24 h P_aCO2_ was significantly reduced and pre-pECLA acidosis was compensated in all subjects. A significant increase of oxygenation index was achieved after 2 h, but remained significantly increased after 24 h only for the surviving cohort. Overall pECLA therapy achieved a stabilization of cardiovascular parameters such as heart ratio, mean arterial pressure, and central venous pressure (Table 2).

**Table 2:** Ventilator settings, blood gas analysis, and hemodynamic parameters before, 2 and 24 h after pECLA initiation

	Before pECLA			2 h after pECLA start			24 h after pECLA start		
Ventilation	all (*n* = 73)	survivor (*n* = 37)	non-survivor(*n* = 36)	all (n = 73)	survivor (n = 37)	non-survivor (n = 36)	all (n = 73)	survivor (n = 37)	non-survivor (n = 36)
peak pressure, mbar	32.7+/− 6.2	32.6+/−7.0	32.6+/− 5.2	30.4+/− 5.4*	31.4+/− 6.1	30.4+/− 4.5*	29.4+/− 4.3^†^	29.1+/− 4.4^†^	29.9+/− 4.3^†^
plateau pressure, mbar	32.7+/−6.2	32.6+/− 7.0	32.6+/− 5.2	30.4+/− 5.4*	31.4+/− 6.1	30.4+/− 4.5*	29.4+/− 4.3^†^	29.1+/− 4.4^†^	29.9+/− 4.3^†^
PEEP, mbar	13.7+/− 4.1	13.6+/− 5.2	12.8+/− 4.7	14.4+/− 3.8	14.9+/− 4.2	13.3+/− 4.6	13.7+/− 3.9	13.7+/− 5.1	12.9+/− 4.7
driving pressure, mbar	19.0+/− 5.6	18.7+/− 6.4	19.0+/− 4.6	16.2+/− 4.6*	16.4+/− 5.1*	16.3+/− 4.4*	15.5+/− 4.2^†^	15.8+/− 4.7^†^	16.2+/− 4.3^†^
T_V_ per kg bodyweight, ml/kg	4.8+/−1.6	4.3+/−2.0	4.5+/− 1.6	4.1+/− 1.2*	3.8+/− 1.3	3.8+/− 0.8*	4.4+/− 1.5	4.3+/− 1.7	3.7+/− 1.2^†^
**Blood gas analysis**									
pH	7.23+/− 0.14	7.24+/− 0.14	7.21+/− 0.15	7.37+/− 0.12*	7.39+/− 0.12*	7.35+/− 0.12*	7.40+/− 0.10†	7.42+/− 0.08^†^	7.37+/− 0.10^†^
P_aCO2_, mmHg	79.4+/−30.6	74.6+/− 25.7	85.9+/− 34.9	51.7+/− 11.0*	49.9+/− 10.4*	53.7+/− 11.5*	48.6+/− 11.6†	45.1+/− 11.4^†‡^	50.9+/− 13.8^†‡^
S_aO2_, %	94.6+/−4.8	94.4+/− 5.7	94.6+/−3.6	95.6+/− 3.1	95.1+/− 6.6	94.9+/− 2.2	96.3+/− 2.4†	96.6+/− 2.1^†^	95.8+/− 2.7
P_aO2_/F_IO2_, mmHg	126+/− 59	132 +/− 75	124+/− 47	107+/− 59*	123+/− 73*^‡^	90+/− 30*^‡^	136+/− 54	151+/− 49^†‡^	120+/− 54^‡^
**Hemodynamics**									
heart rate, bpm	103+/− 22	100+/− 23	104+/− 23	96+/− 21	95+/− 21	99+/− 22	92+/− 19^†^	93+/− 19	91+/− 20^†^
MAP, mmHg	76+/− 14	76+/− 11	77+/− 13	80+/− 14*	81+/− 8*	80+/− 13	83+/− 13^†^	83+/− 13^†^	83+/− 14^†^
CVP, mmHg	16+/− 5	15+/− 4	16+/− 5	15+/− 5	14+/− 5	16+/− 6	14+/− 4	15+/− 4	14+/−5
norepinephrine, μg/kg/min	0.26+/− 0.35	0.22+/− 0.24	0.30+/− 0.44	0.28+/− 0.50	0.21+/− 0.23	0.37+/− 0.69	0.25+/− 0.51	0.17+/− 0.25	0.34+/− 0.69

Abbreviations used for positive end expiratory pressure (PEEP), tidal volume (T_V_), arterial partial pressure of carbon dioxide (P_aCO2_), arterial oxygen saturation (S_aO2_), horowitz index (P_aO2_/F_IO2_), mean arterial pressure (MAP) and central venous pressure (CVP). Data presented as mean ± SD, * *p < 0.05* before vs. 2 h, ^†^*p < 0.05* before vs. 24 h, ^‡^*p < 0.05* alive vs. dead

The results of the multivariable regression analysis are presented in Table 3 demonstrating the correlation between parameters before pECLA and mortality.

**Table 3:** Multivariate analysis of parameters before pECLA start associated with hospital mortality after variable selection

	Multivariate analysis			
Factor	Coefficient	95% Confidence Interval	***r***	***P***
SOFA	−0.044	− 0.073 to − 0.014	−0.24	*< 0.01*
Age	−0.015	0.020 to 1.254	−0.40	*< 0.001*
Immunocompromised status	−0.452	−0.701 to − 0.203	−0.34	*< 0.001*
PaCO_2_ before pECLA	−0.004	−0.007 to − 0.001	−0.19	*< 0.001*
**Overall**			**0.65**	***< 0.001***

**Fig. 1:**
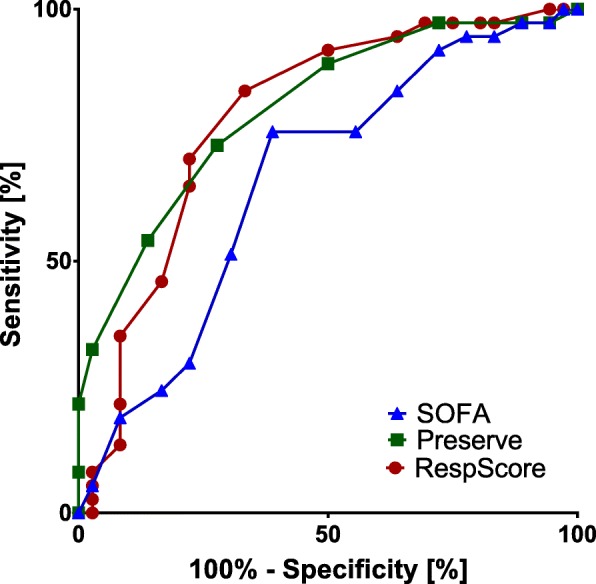
Receiver Operating Characteristic (ROC) curve analysis for RESP, PRESERVE, and SOFA score

For all subjects RESP, PRESERVE and SOFA scores were calculated at initiation of pECLA. Calculated scores for alive versus deceased subjects were 3 ± 1 versus − 1 ± 1 for RESP score (*p < 0.001*), 3 ± 0 versus 6 ± 0 for PRESERVE score (*p < 0.0001*) and 8 ± 1 versus 10 ± 1 for SOFA score (*p < 0.05*). ROC curves (Fig. 1) demonstrated an area under the curve (AUC) of 0.78 for RESP score with a 95% confidence interval (CI) of 0.67–0.89 (*p < 0.001*). PRESERVE score achieved an AUC of 0.80 with 95% CI 0.70–0.90 (*p < 0.0001*) as well as SOFA score an AUC of 0.66 with 95% CI 0.53–0.79) (*p < 0.05*). The calculation of Youden index allowed the definition of a cut-off value for RESP score of 0 (sensitivity 84%, specificity 67%), for PRESERVE score of 4 (sensitivity 73%, specificity 72%) and for SOFA score of 8 (sensitivity 76%, specificity 61%).

## Discussion

With this retrospective study we could demonstrate that RESP and Preserve score are correlating with the mortality of ARDS patients with pECLA. For the first time two specific risk scores were validated for an ECCO_2_R device and were superior to a general organ dysfunction score, the SOFA score. In the past RESP and PRESERVE score were developed and multiple validated for veno-venous ECMO in hypoxemic ARDS.

In the ELSO registry, used for the RESP score definition, only 21% of the subjects had a bacterial pneumonia, and major diagnostic groups were other acute respiratory diagnosis with 28% as well as unspecified with 30%. This origin of ARDS also contributes to the calculated RESP score [1]. Nevertheless in the recently published EOLIA ECMO trial 45% of ARDS subjects suffered from a bacterial pneumonia and 18% from viral pneumonia [19]. In our study, bacterial pneumonia was also the most frequent origin of ARDS with 40% and viral pneumonia was observed in 14%, demonstrating a typical collective of ARDS patients. RESP and PRESERVE score development and validation showed, that age, immunocompromised status, duration of mechanical ventilation, and SOFA score are relevant risk factors for outcome of ECMO [1, 2]. We observed also a significantly younger age, less immunocompromised status, shorter pre-pECLA duration of mechanical ventilation and lower SOFA score in the survivor group (Table 1). There was no direct impact of ARDS etiology to survival rate. Pre- and post-pECLA salvage therapy was not different between survivors and non-survivors. The multivariate analysis of our data revealed also age, SOFA score, immunocompromised status and P_aCO2_ before pECLA as relevant factors for mortality (Table 3). As in former pECLA studies extracorporeal CO_2_ removal allowed an enhanced lung protective ventilation.

The PRESERVE score used a database of 140 ARDS subjects with ECMO to identify risk factors and to generate this score [2]. Subjects presented with a median P_aO2_/F_IO2_ of 53 mmHg (interquartile range 43–60 mmHg), a median P_aCO2_ of 63 mmHg (51–77 mmHg) and a median pH of 7.22 (7.15–7.32) before ECMO. Based on pre-ECMO assessment data of the Extracorporeal Life Support Organization Registry (ELSO) the RESP score was published 2014 using 2355 ECMO cases from 2000 to 2012 [1]. Blood gas analysis revealed similar values before ECMO initiation with a median P_aO2_/F_IO2_ of 59 mmHg (interquartile range 48–75 mmHg), median P_aCO2_ of 56 mmHg (44–73 mmHg) and a median pH of 7.25 (7.15–7.35). In our study, subjects presented with a better oxygenation, indicated by a Horowitz index of 126 ± 59 mmHg, but with a severe respiratory acidosis (P_aCO2_ 79.4 ± 30.6 mmHg and pH 7.23 ± 0.14). Patients with a severe disturbed oxygenation comparable to the PRESERE and RESP validation studies were not suitable for pECLA due to the limited oxygen uptake. These patients were primary connected to veno-venous ECMO. Nine pECLA patients were switched to veno-venous ECMO after further deteriorating oxygenation. Nevertheless, oxygenation and acid base status were more compromised than in the prospective randomized controlled Xtravent study, which evaluated pECLA in combination with an ultraprotective ventilation strategy compared to lung protective ventilation in severe ARDS [20].

ECCO_2_R therapy as arterio-venous pECLA or low-flow veno-venous device seems a promising option to ensure optimized lung protection avoiding further ventilator induced lung injury (VILI) [21] and clinical trials are ongoing [22]. Although there was no leading severe hypoxemia, hospital mortality was 49% in our study compared to 43% in the RESP score study by Schmid et al. [1]. Therefore, in case of extracorporeal carbon dioxide removal a specific risk score seems also useful to identify high-risk patients.

In the PRESERVE and RESP score validation study most of the included patients suffered from severe hypoxemic ARDS [1, 2], whereas only 33% of our subjects had a severe ARDS before pECLA start. In the Berlin definition of ARDS, severity of disturbed oxygenation defines the grade and correlates with mortality [9, 17]. On the other hand severe hypercapnia is independently associated with mortality of ARDS [23]. Therefore, a direct transfer of the RESP and PRESERVE score from ECMO to ECCO_2_R seems not suitable, because patients have different ARDS characteristics with leading hypercapnia and concomitant acidosis but without life-threatening hypoxemia. After positive validation for ARDS patients with leading hypercapnia and ECCO_2_R therapy the established RESP and PRESERVE scores could be used for hypoxic as well as hypercapnic ARDS patients intended for extracorporeal lung support.

Validation of pECLA in our study demonstrated comparable results to other studies analyzing PRESERVE and RESP score for veno-venous ECMO (Table 5). We additionally tested, if a non-specific SOFA score could be an alternative tool to assess the risk profile, but AUC as indicator for accuracy was lower. Nevertheless a SOFA score > 12 represents a risk factor in the PRESERVE score but not in the RESP score. Overall, only the specific scores demonstrated a good diagnostic accuracy for pECLA. Comparing both scores, the PRESERVE score requires less items and as a result seems easier to handle than the RESP score. In conclusion both scores seem suitable for pECLA as ECCO_2_R device.

As mentioned above several studies evaluated RESP and PRESERVE scores for other ECMO populations with differing accuracy and without superiority of one score (Table 4). Survival in the different predefined risk classes demonstrated some inconsistent results but with a generally increasing mortality for a higher risk score (Table 5). Compared to these studies the performance of PRESERVE and RESP was non-inferior for pECLA in our study. Limitations of our study are the retrospective small validation cohort from one ARDS center without additional data from other centers to verify our results, the missing long-term survival data and the restriction to one specific low-flow device for ECCO_2_R. A prospective registry of ECCO_2_R could be able to generate more detailed as well as long-term data. With our retrospective study, PRESERVE and RESP score could be sufficiently validated to identify a high-risk profile before starting an extracorporeal carbon dioxide elimination. Nevertheless, ARDS therapy and especially time of initiation and decision for conventional therapy versus ECCO_2_R or ECMO require clinical assessment and could not be replaced by a simple scoring.

**Table 4:** Comparison of area under the curve of ROC curve with 95% confidence interval (CI) for PRESERVE and RESP score in different validation studies

study	n	treatment	PRESERVE (95% CI)	RESP (95% CI)
Schmidt [2]	140	ECMO	0.89 (0.83–0.94)	NA
Schmidt [1]	2355	ECMO	NA	0.74 (0.72–0.76)
Brunet [15]	41	ECMO	0.69 (0.53–1.87)	0.60 (0.41–0.78)
Kang [16]	99	ECMO	0.64 (0.51–0.77)	0.69 (0.58–0.81)
Klinzing [10]	51	ECMO	0.67 (0.52–0.82)	0.65 (0.50–0.80)
Lee [14]	50	ECMO	0.80 (0.66–0.90)	0.79 (0.65–0.89)
**our cohort**	**73**	**pECLA**	**0.80 (0.70–0.90)**	**0.78 (0.67–0.89)**

**Table 5:** Survival rate in percent as well as absolute number of patients according to risk classes for RESP and PRESERVE score in different studies

**RESP**			**Survival in risk classes in % (n)**				
**study**	**subjects**	**treatment**	**I**	**II**	**III**	**IV**	**V**
Schmidt [1]	2355	ECMO	92 (164)	76 (563)	57 (1033)	33 (449)	18 (146)
Brunet [15]	41	ECMO	NA (0)	50 (6)	43 (14)	20 (5)	50 (2)
Huang [12]	23	ECMO	100 (2)	75 (8)	75 (4)	50 (4)	0 (5)
Hsin [13]	107	ECMO	75 (NA)	68 (NA)	63 (NA)	24 (NA)	38 (NA)
Klinzing [10]	51	ECMO	100 (3)	61 (18)	56 (23)	29 (7)	NA (0)
**our cohort**	**73**	**pECLA**	**55 (11)**	**80 (15)**	**62 (26)**	**15 (13)**	**14 (8)**
**PRESERVE**			**Survival in risk classes in % (n)**				
**study**	**subjects**	**treatment**	**I**	**II**	**III**	**IV**	
Schmidt [2]	140	ECMO	97 (34)	79 (38)	54 (26)	16 (38)	
Brunet [15]	41	ECMO	58 (12)	54 (11)	57 (7)	0 (5)	
Enger [15]	304	ECMO	89 (35)	72 (90)	60 (97)	36 (67)	
Klinzing [10]	51	ECMO	65 (17)	77 (13)	38 (16)	20 (5)	
**our cohort**	**73**	**pECLA**	**100 (12)**	**63 (24)**	**36 (25)**	**17 (12)**	

In our study we focused on pumpless ECLA as ECCO_2_R device, but other veno-venous low-flow ECLA systems are also used for hypercapnic ARDS. For veno-venous devices, there is an ongoing transition from leading decarboxylation to decarboxylation plus oxygenation with increasing blood flow. As RESP and PRESERVE were primary validated for classical high-flow ECMO and now were additionally validated for pECLA as decarboxylation device by our study, we hypothesize that these scoring systems are also suitable for other low-flow ECLA systems. Further investigations of low-flow veno-venous ECCO_2_R could be used to confirm this assumption.

## Conclusions

Performance of RESP and PRESERVE score was at least as good for pECLA as for veno-venous ECMO, the primary validation cohort and this is the first study expanding the scope from high-flow ECMO to an ECCO_2_R therapy. We demonstrated that these risk scores are suitable for ARDS with leading hypercapnia and pECLA additional to severe hypoxemic ARDS with high-flow ECMO.

Both scores, RESP and PRESERVE, but not SOFA score seem suitable to point out the risk profile of ARDS patients with leading hypercapnia and pECLA expanding the scope from ECMO to ECCO_2_R.

## Supplementary information

**Additional file 1.** Definition and calculation of RESP score.

**Additional file 2.** Definition and calculation of the PRESERVE score.

**Additional file 3.** Definition and calculation of the SOFA score.

## Data Availability

The datasets used and analyzed during the current study are available from the corresponding author on reasonable request.

## Abbreviations

ARDS: Acute respiratory distress syndrome
AUC: Area under the curve
CI: Confidence interval
CVP: Central venous pressure
ECCO_2_R: Extracorporeal carbon dioxide removal
ECMO: Extracorporeal membrane oxygenation
ELSO: Extracorporeal Life Support Organization
P_aO2_/F_IO2_: Horowitz index
MAP: Mean arterial pressure
P_aCO2_: Arterial partial pressure of carbon dioxide
pECLA: Pumpless extracorporeal lung assist
PEEP: Positive endexpiratory pressure
PRESERVE: PRedicting dEath for SEvere ARDS on VV-ECMO
RESP: Respiratory ECMO survival score
ROC: Receiver operating characteristic curve
S_aO2_: Arterial oxygen saturation
SOFA: Sequential organ failure assessment score
T_V_: tidal volume
VILI: Ventilator induced lung injury

## References

[1] Schmidt M, Bailey M, Sheldrake J, Hodgson C, Aubron C, Rycus PT (2014). Predicting survival after extracorporeal membrane oxygenation for severe acute respiratory failure. The respiratory extracorporeal membrane oxygenation survival prediction (RESP) score. Am J Respir Crit Care Med.

[2] Schmidt M, Zogheib E, Roze H, Repesse X, Lebreton G, Luyt CE (2013). The PRESERVE mortality risk score and analysis of long-term outcomes after extracorporeal membrane oxygenation for severe acute respiratory distress syndrome. Intensive Care Med.

[3] Combes A, Brodie D, Bartlett R, Brochard L, Brower R, Conrad S (2014). Position paper for the organization of extracorporeal membrane oxygenation programs for acute respiratory failure in adult patients. Am J Respir Crit Care Med.

[4] Terragni P, Maiolo G, Ranieri VM (2012). Role and potentials of low-flow CO (2) removal system in mechanical ventilation. Curr Opin Crit Care.

[5] Bein T, Weber F, Philipp A, Prasser C, Pfeifer M, Schmid FX (2006). A new pumpless extracorporeal interventional lung assist in critical hypoxemia/hypercapnia. Crit Care Med.

[6] Kopp R, Bensberg R, Wardeh M, Rossaint R, Kuhlen R, Henzler D (2012). Pumpless arterio-venous extracorporeal lung assist compared with veno-venous extracorporeal membrane oxygenation during experimental lung injury. Br J Anaesth.

[7] Liebold A, Reng CM, Philipp A, Pfeifer M, Birnbaum DE (2000). Pumpless extracorporeal lung assist - experience with the first 20 cases. Eur J Cardiothorac Surg.

[8] Vincent JL, Moreno R, Takala J, Willatts S, De Mendonca A, Bruining H (1996). The SOFA (sepsis-related organ failure assessment) score to describe organ dysfunction/failure. On behalf of the working group on sepsis-related problems of the European Society of Intensive Care Medicine. Intensive Care Med.

[9] Bellani G, Laffey JG, Pham T, Fan E, Brochard L, Esteban A (2016). Epidemiology, patterns of care, and mortality for patients with acute respiratory distress syndrome in intensive care units in 50 countries. JAMA.

[10] Klinzing S, Wenger U, Steiger P, Starck CT, Wilhelm M, Schuepbach RA (2015). External validation of scores proposed for estimation of survival probability of patients with severe adult respiratory distress syndrome undergoing extracorporeal membrane oxygenation therapy: a retrospective study. Crit Care.

[11] Enger T, Philipp A, Videm V, Lubnow M, Wahba A, Fischer M (2014). Prediction of mortality in adult patients with severe acute lung failure receiving veno-venous extracorporeal membrane oxygenation: a prospective observational study. Crit Care.

[12] Huang L, Li T, Xu L, Hu XM, Duan DW, Li ZB (2016). Performance of multiple risk assessment tools to predict mortality for adult respiratory distress syndrome with extracorporeal membrane oxygenation therapy: an external validation study based on Chinese single-center data. Chin Med J.

[13] Hsin CH, Wu MY, Huang CC, Kao KC, Lin PJ (2016). Venovenous extracorporeal membrane oxygenation in adult respiratory failure: scores for mortality prediction. Medicine (Baltimore).

[14] Lee S, Yeo HJ, Yoon SH, Lee SE, Cho WH, Jeon DS (2016). Validity of outcome prediction scoring Systems in Korean Patients with severe adult respiratory distress syndrome receiving extracorporeal membrane oxygenation therapy. J Korean Med Sci.

[15] Brunet J, Valette X, Buklas D, Lehoux P, Verrier P, Sauneuf B (2017). Predicting survival after extracorporeal membrane oxygenation for ARDS: an external validation of RESP and PRESERVE scores. Respir Care.

[16] Kang HR, Kim DJ, Lee J, Cho YJ, Kim JS, Lee SM (2017). A comparative analysis of survival prediction using PRESERVE and RESP scores. Ann Thorac Surg.

[17] The Ards Definition Task Force (2012). Acute respiratory distress syndrome: the Berlin definition. JAMA.

[18] Kopp R, Kuhlen R, Max M, Rossaint R (2002). Evidence-based medicine in the therapy of the acute respiratory distress syndrome. Intensive Care Med.

[19] Combes A, Slutsky AS, Brodie D (2018). ECMO for severe acute respiratory distress syndrome. N Engl J Med.

[20] Bein T, Weber-Carstens S, Goldmann A, Muller T, Staudinger T, Brederlau J (2013). Lower tidal volume strategy ( approximately 3 ml/kg) combined with extracorporeal CO2 removal versus 'conventional' protective ventilation (6 ml/kg) in severe ARDS: the prospective randomized Xtravent-study. Intensive Care Med.

[21] Beitler JR, Sands SA, Loring SH, Owens RL, Malhotra A, Spragg RG (2016). Quantifying unintended exposure to high tidal volumes from breath stacking dyssynchrony in ARDS: the BREATHE criteria. Intensive Care Med.

[22] McNamee JJ, Gillies MA, Barrett NA, Agus AM, Beale R, Bentley A (2017). Protective vEntilation with veno-venouS lung assisT in respiratory failure: a protocol for a multicentre randomised controlled trial of extracorporeal carbon dioxide removal in patients with acute hypoxaemic respiratory failure. J Intensive Care Soc.

[23] Nin N, Muriel A, Penuelas O, Brochard L, Lorente JA, Ferguson ND (2017). Severe hypercapnia and outcome of mechanically ventilated patients with moderate or severe acute respiratory distress syndrome. Intensive Care Med.

