# Supplementary material for: Validation of RESP and PRESERVE score for ARDS patients with pumpless extracorporeal lung assist (pECLA)
Source: BMC Anesthesiol. 2020 May 2;20:102. doi: 10.1186/s12871-020-01010-0 (PMC7195797; doi:10.1186/s12871-020-01010-0)
Supplement: Supplementary file 1 — Additional file 1. Definition and calculation of RESP score. [file 12871_2020_1010_MOESM1_ESM.docx]

**Additional file 1:** Definition and calculation of RESP score.

| Age (years) | 18-49 | **0** |
| --- | --- | --- |
|  | 50-59 | **−2** |
|  | ≥60 | **−3** |
| Immunocompromised status | | **−2** |
| Mechanical ventilation prior to initiation of ECMO | <48 hours | **3** |
|  | 48 hours to 7 days | **1** |
|  | >7 days | **0** |
| Acute respiratory diagnosis group (select only one) | Viral pneumonia | **3** |
|  | Bacterial pneumonia | **3** |
|  | Asthma | **11** |
|  | Trauma and burn | **3** |
|  | Aspiration pneumonitis | **5** |
|  | Other acute respiratory diagnoses | **1** |
|  | Nonrespiratory and chronic respiratory diagnoses | **0** |
| Central nervous system dysfunction | | **−7** |
| Acute associated (nonpulmonary) infection | | **−3** |
| Neuromuscular blockade before ECMO | | **1** |
| Nitric oxide use before ECMO | | **−1** |
| Bicarbonate infusion before ECMO | | **−2** |
| Cardiac arrest before ECMO | | **−2** |
| P_aCO2_ (mmHg) | <75 | **0** |
|  | ≥75 | **−1** |
| Peak inspiratory pressure (cmH_2_O) | <42 | **0** |
|  | ≥42 | **−1** |
| **Total Score** |  | **-22 to +15** |
|  |  |  |
| **Hospital Survival by Risk Class** | | |
| **Risk Class** | Survival Rate | **Score** |
| **I** | 92% | **≥6** |
| **II** | 76% | **3 to 5** |
| **III** | 57% | **-1 to 2** |
| **IV** | 33% | **-5 to -2** |
| **V** | 18% | **≤ -6** |
