# Supplementary material for: Validation of RESP and PRESERVE score for ARDS patients with pumpless extracorporeal lung assist (pECLA)
Source: BMC Anesthesiol. 2020 May 2;20:102. doi: 10.1186/s12871-020-01010-0 (PMC7195797; doi:10.1186/s12871-020-01010-0)
Supplement: Supplementary file 2 — Additional file 2. Definition and calculation of the PRESERVE score. [file 12871_2020_1010_MOESM2_ESM.docx]

**Additional file 2:** Definition and calculation of the PRESERVE score.

| Age, years | <45 | **0** |
| --- | --- | --- |
|  | 45-55 | **2** |
|  | >55 | **3** |
| BMI > 30 kg/m² | | **-2** |
| immunocompromised | | **2** |
| SOFA > 12 | | **1** |
| mechanical ventilation > 6 days | | **1** |
| no prone positioning before ECMO | | **1** |
| PEEP < 10 cm H_2_O | | **2** |
| plateau pressure >30 cm H_2_O | | **2** |
| **Total Score** | | **-2 to 12** |
|  | |  |
| **6 Month Survival by Risk Class** | | |
| **Risk Class** | Survival Rate | **Score** |
| **I** | 97% | **-2 to 2** |
| **II** | 79% | **3 to 4** |
| **III** | 54% | **5 to 6** |
| **IV** | 16% | **7 to 12** |
