# Supplementary material for: Validation of RESP and PRESERVE score for ARDS patients with pumpless extracorporeal lung assist (pECLA)
Source: BMC Anesthesiol. 2020 May 2;20:102. doi: 10.1186/s12871-020-01010-0 (PMC7195797; doi:10.1186/s12871-020-01010-0)
Supplement: Supplementary file 3 — Additional file 3. Definition and calculation of the SOFA score. [file 12871_2020_1010_MOESM3_ESM.docx]

**Additional file 3:** Definition and calculation of the SOFA score.

| P_aO2_/F_IO2_, mmHg | <400 | 1 |
| --- | --- | --- |
|  | <300 | 2 |
|  | <200 | 3 |
|  | <100 | 4 |
| Platelets, G/l | <150 | 1 |
|  | < 100 | 2 |
|  | < 50 | 3 |
|  | <20 | 4 |
| Bilirubin, mg/dl | 1.2-1.9 | 1 |
|  | 2.0-5.9 | 2 |
|  | 6.0-11.9 | 3 |
|  | > 12.0 | 4 |
| Mean arterial pressure | MAP< 70 mmHg | 1 |
|  | dopamine > 5 µg/kg/min or dobutamine | 2 |
|  | epinephrine/norepinephrine ≤ 0.1 µg/kg/min | 3 |
|  | epinephrine/norepinephrine > 0.1 µg/kg/min | 4 |
| Creatinine or urine output | 1.2-1.9 mg/dl | 1 |
|  | 2.0-3.4 mg/dl | 2 |
|  | 3.5-4.9 mg/dl or < 0.5 l/d | 3 |
|  | > 5.0 mg/dl or < 0.2 l/d | 4 |
| Glasgow Coma Scale | 13-14 | 1 |
|  | 10-12 | 2 |
|  | 6-9 | 3 |
|  | < 6 | 4 |
| **Total Score** |  | **0 to 24** |
